# Flexible learning, rather than inveterate innovation or copying, drives cumulative knowledge gain

**DOI:** 10.1126/sciadv.aaz0286

**Published:** 2020-06-05

**Authors:** Elena Miu, Ned Gulley, Kevin N. Laland, Luke Rendell

**Affiliations:** 1 Centre for Social Learning and Cognitive Evolution, School of Biology, University of St Andrews, St Andrews KY16 9TH, UK.; 2 School of Human Evolution and Social Change and Institute of Human Origins, Arizona State University, Tempe, AZ 85287, USA.; 3 MathWorks, Natick, MA 01760, USA.

## Abstract

Human technology is characterized by cumulative cultural knowledge gain, yet researchers have limited knowledge of the mix of copying and innovation that maximizes progress. Here, we analyze a unique large-scale dataset originating from collaborative online programming competitions to investigate, in a setting of real-world complexity, how individual differences in innovation, social-information use, and performance generate technological progress. We find that cumulative knowledge gain is primarily driven by pragmatists, willing to copy, innovate, explore, and take risks flexibly, rather than by pure innovators or habitual copiers. Our study also reveals a key role for prestige in information transfer.

## Teaser

Cumulative cultural evolution is driven by pragmatic individuals who know when to copy, innovate, explore, and take risks flexibly.

## INTRODUCTION

Culture is responsible for the behavioral diversity that has led to our species’ remarkable adaptability and ecological success (*1*, *2*). At the heart of culture lies social learning—learning influenced by contact with other individuals (*3*)—which is used by an extensive variety of species. Only in humans, however, do we see compelling evidence for the buildup of socially transmitted information over multiple rounds of innovation and social learning, often into complex multicomponent functional solutions, leading to tools, products, and knowledge that no one individual could have invented alone (*2*, *4*, *5*).

Strong evidence suggests that individuals should use social learning selectively according to strategies that guide how, what, and under what circumstances they copy others, and when they rely on their own experience (*6*–*11*). Recent studies show that humans exhibit consistent individual differences in the rates of using either social or asocial information in decision-making (*12*–*16*), with these preferences consistent across time and contexts (*13*), and linked to personality traits in both adults (*17*) and children (*18*). Such individual differences in social-information use have profound implications for the way researchers conceptualize and model social learning. In particular, there has been little research thus far on how variation in learning strategies between and within individuals could affect the processes underlying cumulative cultural evolution.

Here, we analyze a unique large-scale dataset to investigate, in a cumulative cultural evolution setting, whether and how individual differences in learning generate collective progress. The dataset arises from a series of collaborative online programming competitions organized by the MathWorks software company over the course of 14 years (*19*). Each contest involved participants attempting to craft and improve solutions to a set of NP-complete computer coding challenges (*20*). Such challenges do not have an exact solution, which allows open-ended improvement, as typically characteristic of cumulative cultural evolution. This exclusive dataset provides a rare opportunity to isolate the causes of technological progress in a setting that approaches real-world complexity.

Complex cultural systems, often characterized by opaque links between cultural traits and payoffs, require individuals to use effective heuristics to guide their learning. One cue thought to be particularly important in human societies is prestige, defined as high status or influence typically related to higher competence in valued domains of activity (*21*). In complex contexts when direct observation of payoffs is difficult, watching how much other individuals defer to, attend to, or copy a model can provide an efficient proxy for that model’s information quality (*22*, *23*). Prestige can extend across domains, for example, being perceived as a successful yam grower might still increase the probability that an individual’s fishing techniques would be copied (*4*).

The complex interactions characterizing cumulative cultural evolution provide an ideal context for such “prestige bias” to emerge. Repeated interactions between individuals in a challenging environment characterized by hard problems allow individuals to create reputations that are used to guide the copying of beneficial traits. In the aforementioned programming contests, once an individual submitted a valid entry, it became public, making its code accessible to other participants, along with its score and the author’s chosen username. Over time, some individuals took part in more than one contest, which allowed the potential to build reputation and influence across contests.

Here, we show that the successful individuals that drive cumulative improvements in the programming contests are neither habitual innovators nor inveterate copiers, but rather mixed-strategy pragmatists, willing to copy, innovate, explore, and take risks flexibly. We further demonstrate that superior performance in contests allows players to generate reputations that are used by other players as a cue to guide social learning above and beyond the effect of payoff bias, both within and across contests.

## RESULTS

### Variation between and within individuals

We analyzed data from 19 online programming competitions organized by MathWorks from 1998 until 2012 (*19*). Overall, we had data from 1964 unique participants from 19 contests, with an average of 136 participants per contest, some of whom took part in more than one contest, and collectively submitted a total of 45,793 valid entries. We grouped submitted entries according to the participant that submitted them (henceforth called “contestant”) both within each contest and, where possible, across different contests. Each contestant was thus responsible for a collection of entries, which can be characterized in terms of activity (the total number of entries submitted to the contest), novelty (similarity to the entry with the current best score—as there is substantial copying taking place in the contest, this similarity is an unbiased, relative measure of how much an entry is deviating from the current population consensus), and performance (whether the entry became, on submission, a leader in its contest, i.e., whether it achieved the best score at the time of its submission). Each individual contestant could thus be characterized by a number of entries, a distribution of leader similarities, and a distribution of performance measures for every entry they had submitted. To begin with, for simplicity, we analyzed each contest as independent and assumed that all contestants were distinct (i.e., contestants were not linked across contests; see below for an analysis considering the same individuals participating in multiple contests). This means that each contestant had an associated activity, novelty, and performance measure for each contest in which they participated.

We found that individuals differed widely in their activity, use of novelty, and performance. Activity ranged from those who only submitted one entry to very active, very exploratory individuals who returned a wide range of raw scores. The number of entries per contestant was approximately exponentially distributed in all contests, with 30% of contestants submitting only 1 entry in the entire contest, 60% submitting 5 or fewer, and less than 1% submitting >50 entries (fig. S1). Of all participants to all contests, 22% submitted at least one entry that took the lead, and 14% did this more than once. The average number of entries per leading contestant was 10 times larger than the average number of entries per nonleading contestant. Activity was therefore strongly linked to performance at the individual level (fig. S2). However, the variation in activity levels among leading contestants indicates that high activity was not necessary for a participant to be able to take the lead—8% of leading contestants submitted only a single entry, while 16% submitted less than five (fig. S2).

**Fig. 1:**
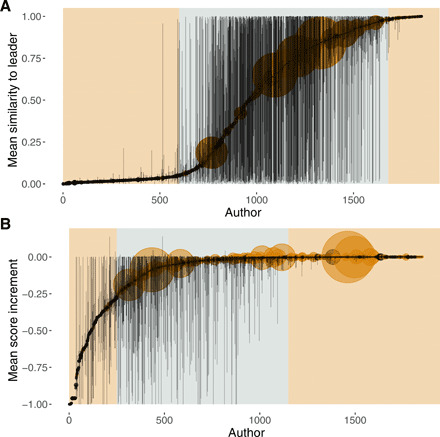
Between- and within-individual variation in similarity and performance. Similarity to current leader (**A**) and score increment (**B**)—average values with bars spanning the range of the distribution. The colored circles indicate leading contestants (i.e., contestants who submitted at least one entry that improved the overall score at the time of its submission), and the size of the circles is proportional to the total number of entries submitted by each contestant. The shaded panels in (A) indicate a visual split of participants into mavericks (left), copiers (right), and flexible users (middle) based on how they make use of social learning. The shaded panels (B) indicate a visual split between poorly performing contestants (left), contestants who are variable in performance (middle), and consistently good performers (right).

The novelty results show that this between-individual variation extends to how individuals used social learning in their solutions. Some contestants were very conservative and preferred to keep their entries “safe” through solely copying the current leader, while other contestants were relatively adventurous, submitting entries that varied in their novelty (Fig. 1A). However, contestants did not display a bimodal distribution in use of copying or introduction of novelty, but rather could be broadly split into three groups, albeit on a continuous distribution: (i) a surprisingly large number of contestants who only submitted entries with low similarity to the current leader, a group that we term “incurable mavericks,” who barely ever took the lead (Fig. 1A, left section); (ii) an intermediate group whose entries ranged from zero similarity to very close copies of the current leader, termed “occasional mavericks,” who were the most likely group to take the lead (Fig. 1A, middle section); and (iii) a smaller group whose entries were always very similar to the current leader, termed “extreme conservatives,” who, again, rarely took the lead (Fig. 1A, right section). Most leading contestants and the most active contestants lie toward the copying end of this spectrum.

**Fig. 2:**
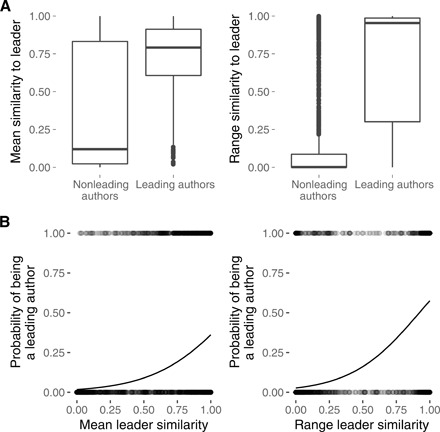
The link between similarity and performance. (**A**) Distributions of average leader similarities and distribution of leader similarity ranges for all nonleading and leading contestants. (**B**) Probability of a contestant becoming a leading contestant as a function of mean leader similarity and the range of the leader similarity distribution, as predicted by the generalized linear mixed model.

There was also considerable between-individual variation in terms of performance (Fig. 1B), with participants again split into three groups: a number of contestants who displayed very little variation in scores relative to the current leader and who often took the lead but more often than not only submitted one or two entries (Fig. 1B, right section), a group of contestants who showed variation in performance but tended to take the lead (Fig. 1B, middle section), and a final group of contestants who varied in their scores but showed poor performance on average (Fig. 1B, right section). Leading contestants use social information in a notably different manner to other contestants (Fig. 2). We split participants into leading contestants (i.e., contestants who submitted at least one leading entry that beat the current best in the contest, in at least one contest) and nonleading contestants (who never submitted any entry in any contest that beat the current best leader). To test whether the way individuals used social information affected their performance, we fitted a generalized linear mixed model with a binomial error distribution that predicted whether an individual was a leading contestant or not as a function of the mean and the range of the distribution of similarities between that individual’s submissions and the current leader at the time of submission, to ask whether more or less innovation in terms of solutions was beneficial.

According to the generalized mixed linear model (GLMM), the probability of a contestant introducing leading entries increased with a higher mean similarity to the current leader, but was also correlated with a higher range of the distribution of similarities between each entry submitted by the author and the current leader (i.e., the variation of solutions submitted by the author; Fig. 2B and Table 1). For every one unit increase in mean similarity, the log odds of taking the lead increasd by 3.488 (i.e., the odds increased by a factor of 31). Therefore, both between-individual variation (i.e., how much copying a contestant engages in on average, as measured by the average similarity to the current leader) and within-individual variation (i.e., how variable the solutions submitted by each contestant are, as measured by the range of the distribution of similarities to the leader for each individual) are predictors of individual performance. Leading contestants, who were almost always occasional mavericks, were more similar to the current leader, on average, than nonleading contestants. However, leading contestants also showed considerable flexibility in their behavior, being substantially more variable in their use of social and asocial information than nonleading contestants. Leading contestants deviated more often from the status quo than extreme conservatives, while (unlike incurable mavericks) still sometimes working on variations of the current leading solution and scoring consistently better than nonleaders even with their nonleading entries (fig. S3). Results from the continuous version of this model confirm our findings (table S1 and fig. S4).

**Table 1:** The effect of social learning and exploration on whether the contestants ever took the lead or not. Results from GLMM: LeadingContestant ~ MeanScoreDifference + MeanLeaderSimilarity + RangeLeaderSimilarity + (1|Contest). Predictors are standardized—similarity ranges theoretically between 0 and 1 and score difference between −1 and 1.

**Fixed** **effects**	**Estimate**	**SE**	***z* value**	**95% confidence** **interval**
(Intercept)	−4.919	0.342	−14.37	−5.622 to −4.275
Mean score difference	2.752	0.893	3.08	1.096–4.621
Mean leader similarity	3.488	0.34	10.24	2.844–4.182
Range leader similarity	3.907	0.215	18.14	3.498–4.344

### Influence

**Fig. 3:**
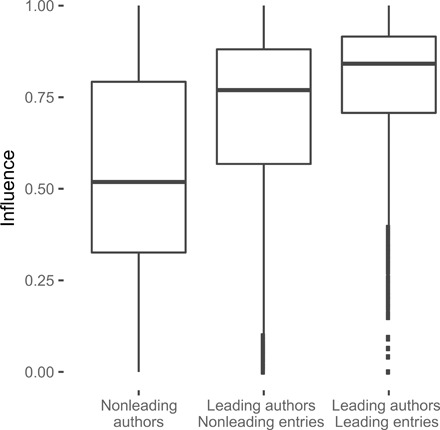
Leaders have higher influence. Entry-level influence distribution for entries submitted by nonleading contestants, nonleading entries submitted by leading contestants, and leading entries submitted by leading contestants.

We devised a measure, which we call “influence,” that captures how much of an entry a population picked up following the entry’s submission. Influence is broadly calculated as a normalized version of the average similarity between an entry and subsequent entries submitted by other contestants in that contest, thus capturing how much of an entry is reflected following entries, while controlling for self-similarity (fig. S5). Leading contestants were also copied more (i.e., they had, on average, higher influence) than nonleading contestants, through both their leading and their nonleading entries (Fig. 3). Leading entries had higher influence than nonleading entries overall, but even nonleading entries submitted by leading contestants had higher influence than entries submitted by nonleading contestants (Table 2), even when we control for score difference. For instance, a nonleading entry submitted by a leading contestant had a 0.135-point increase in influence compared to a nonleading entry submitted by a nonleading contestant. If the entry was also leading, this added another 0.175-point increment. This was in addition to the increase in influence due to higher increment. Notably, leading contestants submitted entries that had a higher influence on other participants, even when those entries were not the best available to copy, and even when variation in actual score was accounted for. This demonstrates that a prestige effect was taking place in the contests, with contestants who manage to take the lead at least once forming reputations that influenced how others copied.

**Table 2:** Influence within contests for entries that did/did not take the lead, submitted by contestants who did/did not take the lead. Results for fixed effects from linear mixed model: Influence ~ LeaderGroup + Increment + (1|Contest/Contestant). The top row represents the baseline, entries that neither led nor were submitted by contestants who were ever leaders. The following two rows indicate additive effects relative to the baseline for nonleading entries submitted by leading contestants and leading entries submitted by leading contestants. The last row indicates the relationship between influence and performance as measured by score increment (standardized between −1 and 1).

**Fixed effects**	**Estimate**	**SE**	***t* value**	**95% confidence** **interval**
(Intercept)	0.578	0.025	22.96	0.527–0.629
Nonleading contestant				
Nonleading entry				
Leading contestant	0.135	0.010	12.77	0.114–0.155
Nonleading entry				
Leading contestant	0.175	0.010	15.96	0.153–0.197
Leading entry				
Score increment	0.314	0.007	44.65	0.3001–0.328

Crucially, this effect extended across contests (fig. S6). Overall, again, leading entries had significantly higher influence than nonleading entries, and so did nonleading entries that had been submitted by a contestant who managed to take the lead in the same contest. Leading entries submitted by a contestant who was a leader in the current contest had, on average, 0.243 higher influence than nonleading entries submitted by a contestant who was never a leader, but even nonleading entries submitted by a contestant who had taken the lead in the contest had, on average, 0.217 higher influence (Table 3). More surprisingly, this prestige effect held even for entries that did not take the lead, submitted by contestants who did not become leaders in the focal contest, but had taken the lead in a different contest (Table 3), which achieved 0.120 higher influence than the baseline, nonleading entries submitted by contestants who never led. This was true while controlling for payoff bias, i.e., mean performance overall—for every unit increase in mean score increment, influence increased by 0.284 units. This shows that cross-contest individual behavior was significantly related to entry-level measures of influence, indicating that consistent individual characteristics affected how entries were copied, in line with the expectations if prestige effects were forming across contests through repeated participation.

**Table 3:** Influence across contests for leading/nonleading entries submitted by contestants who never took the lead, who took the lead in the same contest the entry was submitted in, or who took the lead in a different contest. Results for fixed effects from linear mixed model: Influence ~ LeaderGroup + Increment + (1|Contestant/Contest). The first row indicates the intercept: nonleading entries submitted by nonleading contestants who never took the lead in any other contests. The following rows indicate the additional effect corresponding to each factor level indicated—bold indicates leading (either entry, contestant, or contestant in a different contest). The last row indicates the relationship of influence with performance, measured here as score increment.

**Fixed effects**	**Estimate**	**SE**	***t* value**	**95%** **confidence** **interval**
(Intercept)	0.483	0.024	19.64	0.435–0.531
Nonleading entry				
Nonleading contestant				
Not leading elsewhere				
Nonleading entry	0.120	0.032	3.67	0.056–0.184
Nonleading contestant				
**Leading** **elsewhere**				
Nonleading entry	0.217	0.057	3.78	0.105–0.329
**Leading** **contestant**				
Not leading elsewhere				
Nonleading entry	0.242	0.030	7.87	0.182–0.303
**Leading** **contestant**				
**Leading** **elsewhere**				
**Leading entry**	0.243	0.062	3.90	0.121–0.365
**Leading** **contestant**				
Not leading elsewhere				
**Leading entry**	0.283	0.031	9.11	0.222–0.344
**Leading** **contestant**				
**Leading** **elsewhere**				
Score increment	0.284	0.008	32.28	0.267–0.302

## DISCUSSION

In a cumulative cultural evolution setting with real-world task complexity, we have shown that individual differences in reliance on social and asocial learning give rise to considerable variation in performance. Analysis of individual-level patterns of entry novelty did not indicate a dichotomous split between individuals who preferred copying and those who preferred innovation, but rather a continuous spectrum, in which individuals varied not only in their proclivity to copy and/or innovate but also in how much within-individual variation (i.e., exploration across entries) they displayed. Notably, the spectrum of the individual reliance on social learning had long tails formed by individuals with relatively pure “always innovate” and “always copy” approaches, who had relatively low success overall. The best-performing individuals occupied the center ground, mixing a balance of copying the leader with their own innovation and exhibiting flexibility and exploration in achieving this balance. Our results suggest that, to succeed, it is not enough to innovate alone, or solely to copy uncritically, but rather, individuals must strike a balance between the two. Successful individuals are pragmatists, willing to copy, innovate, explore, and take risks.

Previous work acknowledges and occasionally focuses on between-individual variation in social-information use (*9*, *15*, *24*), sometimes identifying factors that could explain this variation, such as confidence (*9*), intelligence quotient (*25*), or age (*26*). For instance, in a dataset of 60 years of opening moves in the game of Go, Beheim *et al.* (*27*) found both individual variation in social-information use (some players copy more than others), as well as cultural variation (players from certain countries copy more than others). Modeling work has shown that a mix of innovation and social learning can be beneficial, at both the population and individual level (*6*, *11*, *28*), and improvement is maximized by a careful blend of exploration and copying (*29*, *30*). The literature less often discusses within-individual variation in social learning and how it is linked to population-level improvement. Morgan *et al.* (*9*) show that individuals flexibly adjust their reliance on social information over time, as they gain confidence in the task, and Toelch *et al.* (*31*) show that individuals change their reliance on innovation when presented with social performance cues. Such findings are indicative of growing evidence that humans implement learning strategies flexibly (*32*). We extend these findings to show that not only do individuals use social information flexibly but also this flexibility is adaptive in the sense of being associated with successful performance: The best-performing individuals are those that most effectively navigate the trade-off between innovation and social learning.

Within our current framework, it is not immediately obvious how this trade-off is negotiated, or even how to predict accurately how good ideas are generated. Individual preferences for copying versus exploration can be explained in terms of both perceived expected payoffs and built-in proclivities for either type of learning. The structure of the scoring system allows for better scores either through algorithmic improvement or through speeding up the code, which means that copying is a safe strategy and individuals who are not especially proficient coders will typically receive higher payoffs from copying than innovating. Nonetheless, we see evidence of poor performers who stick exclusively to innovating and refuse to copy, suggesting a personal preference, manifest independent of payoff. Given the substantial search space of existing solutions, the fact that entries tend to have high similarity to the current leader is not surprising, as copying the leader is a quick heuristic for reducing the space and focusing on proven solutions. The fact that new leaders are both similar to current leaders and more exploratory could be interpreted as leaders being good at innovating from a starting point of the current best solution, although studies show that a degree of randomness can aid exploratory search (*33*, *34*). However, our data imply that both conservatism and exploration play a role in effective innovation. We have shown in previous work that many leading entries were very similar to the current leader, but a handful were very different, yet associated with higher improvement (*19*). The latter generated large innovative leaps that triggered the population to adopt this new solution, which was then optimized through small modifications. Here, we show that the individuals responsible for these crucial entries rarely worked alone and also participated in the tweaking process. Overall, leaders showed a higher level of engagement than nonleaders, perhaps symptomatic of relevant personal motivators (interest, expertise, and perseverance).

Prestige effects are expected to emerge where there is a correlation between status and performance (*21*). According to participant accounts, introducing an entry that takes the lead is a highly sought-after prize, which suggests that reputation is a valued commodity in these contests. Moreover, participants remember good players from previous contests and pay attention to their submissions. Our study provides clear evidence that leaders had more influence on the patterns of solutions in the population than nonleaders, even when their entries did not take the lead. This effect extended across contests such that individuals who had proved successful had influence even in contests in which they never took the lead. Modeling the influence of leaders while controlling for the individual performance of each entry allowed us to establish whether leaders had higher influence merely as a result of submitting generally better entries or whether leadership genuinely creates a reputational effect. The analysis confirms genuine prestige effects in the copying of leaders. This prestige effect held up across contests, suggesting that an individual’s reputation builds in the MATLAB contest world independently of the specific challenge, perhaps serving as a heuristic used to reduce the overwhelming search space. This is in line with Henrich and Gil-White’s theory (*21*), suggesting that prestige can be a useful tool in the face of uncertainty, even when that uncertainty is not a result of lack of success information, but rather an excess of it. However, such effects are still reliant on general programming expertise, as opposed to, say, a gifted footballer promoting a brand of clothing, and it remains to be established how widely this cross-domain influence extends.

We have investigated how individual-level use of social information contributes to technological progress in a cumulative cultural evolution microcosm, where cultural artifacts are incrementally improved over time through modifications by multiple individuals. We studied what humans do when unguided or unprompted, confirming and extending results from theoretical models and small experiments in a large-scale realistic setting. Although there were no experimental interventions in this study, we can nonetheless draw clear inferences about the factors that shape cumulative cultural change. Our results suggest that overt attempts to maximize cumulative cultural adaptation require populations consisting of many individuals exhibiting “leader” qualities (i.e., exploring and flexibly switching between social and asocial information). We have also shown that prestigious individuals have a disproportionate influence on cultural transmission, a finding that implies that performance increments may be achieved through coupling prestige with superior solutions. We note, however, that prestige bias need not speed up cumulative cultural evolution, if this means that good solutions introduced by nonprestigious individuals are hindered from spreading through the population. While our study system might mimic patterns of improvement in some contemporary scenarios—today’s business world, for example—it is limited in its generality. For instance, many cultural adaptation scenarios do not involve the level of competition or transparency manifest here. Further realistic studies of such phenomena are needed to establish the generality of our findings.

Consistent interindividual variation in behavior has been a focus in behavior studies for over a decade, sometimes controversially (*35*). Our study contributes to the growing expectation that differences between individuals, and groups, in their approach to learning will have important effects on the patterns of cultural evolution (*36*).

However, our findings also draw attention to within-individual flexibility in the use of social and asocial information, suggesting that not only the predilection to use social information but also the contexts in which humans copy could be learned. Here, any assumption that social learning strategies are not learned could underestimate the speed of response to environmental variation (*36*), as well as the patterns of change of these social learning strategies (*32*), and the vulnerability to the propagation of maladaptive traits (*37*). Our study implies that flexibility in learning is a key ingredient for successful innovation.

Last, our study provides compelling evidence for prestige bias. To date, little empirical work has focused on the importance of prestige bias [see (*38*) for a review of the existing literature], but the complex cumulative cultural evolution microcosm provided by our dataset provides a useful framework for studying this learning mechanism in a naturalistic setting. Why prestige effects should be so potent is unclear, but plausibly, this bias has been co-opted as part of norm psychology, a psychological suite of traits evolved to support cultural evolution (*39*), and is used even in the presence of more effective learning mechanisms. If the effect of prestige is manifest even in the presence of a clear cue of success, then our findings suggests that prestige could play an even more prominent role in human social learning contexts in which payoffs are opaque.

## MATERIALS AND METHODS

We analyzed data from 19 online programming competitions organized by MathWorks, the company that produces MATLAB, from 1998 to 2012 (*19*, *40*). Each contest involved the organizers proposing an NP-complete problem (traveling salesman–type constraint problems; see the Supplementary Materials for an example) and participants submitting solutions to it, in the form of MATLAB code. Each entry was submitted to an online platform and evaluated automatically. Once submitted, each entry, along with its score, submitting author, and time of submission, was freely available on the website for all the other participants to access and copy. Participants could only learn their score by submitting an entry. Prizes were nominal (e.g., a MATLAB T-shirt), and participants competed mainly for reputation. Participants were incentivized with small intermediate awards like daily leader and highest improvement in a day. Both these intermediary prizes and the final winner were highly sought-after accolades. The contest attracted programmers that varied in their skill level and engagement with MATLAB, from beginners to engineers and academics who use MATLAB proficiently in their professional life.

Our dataset consisted of 1964 participants from 19 contests, with an average of 136 participants per contest, some of whom took part in more than one contest, and collectively submitted a total of 45, 793 valid entries. Participants submitted an average of 21 entries each, but with very large variation between participants, ranging between 1 and 1502 entries submitted. Of the total of 1964 individual participants, 83% participated in only one contest, and the average number of contests participated in was 1.34, with 2 participants competing in 14 of the 19 contests we studied.

Throughout the week of each contest, participants were allowed to submit as many solutions as they wanted through an online interface, which resulted in numerous participants submitting multiple entries. The participants were identified using an identification number that was linked to a MathWorks account that they themselves created and that was needed to submit entries to the contest. Individuals were not forbidden from creating multiple accounts if they wished to do so, but we have reason to believe, based on online communication between participants, that most did not and, because this would have required substantial effort (e.g., creating a new account, linked to a new email address), we expect that this was not a major confounding factor in this analysis.

The score of each entry was a function of its effectiveness on the task, the speed of execution, and code complexity, measured using McCabe’s cyclomatic complexity (*41*), such that improving an entry could be achieved by improving the success of the algorithm and/or the speed of execution, and/or reducing its complexity (the latter could be achieved without considerable programming proficiency). Entries were disqualified if they exceeded execution time or length limits, and the winner was the entry with the lowest score at the end of the week.

### Characterizing individual variation through activity, novelty, and performance

We characterized individual variation through three principal metrics that we term “activity,” “novelty,” and “performance.” The analysis included only valid entries, which followed the contest guidelines and received a score (if an entry contained a bug that stopped execution, it was not valid and did not receive a score). Some of the contests included a period of “darkness” in the first 2 days, in which contestants only had access to their own entries, in an attempt to encourage individual exploration. To compare accurately across contests, in our analysis, we included only data from the third day onward for all contests.

Activity was measured as the total number of entries submitted in a contest. At the individual level, activity is an indirect measure of motivation—we expected that more motivated, more interested players would submit more entries throughout the contest.

Novelty is inversely related to social learning, and hence, this measure allowed us to quantify and investigate individual differences in both reliance on social learning and innovation, as well as link these factors to performance and thereby establish their adaptive value. To measure novelty, we first used similarity to the current leader as an index of copying. We have shown elsewhere (*19*) that solutions quickly become very complex, which incentivizes participants to copy the current leader (i.e., the entry with the best score at a set time) substantially and tweak that leading solution instead of submitting completely original entries. As a result, populations converged on similar solutions over each contest. Entries are much more similar to the current leader than to any other entries, and although this similarity might not indicate direct copying but rather could be mediated through third entries that copied the current leader, it is nonetheless a robust measure of how much an individual is deviating from the population consensus and, reciprocally, a measure of how much novelty they are introducing. Although we could have used raw proportion of new lines introduced into the contest as a straightforward measure of novelty, this would be a biased measure—there is much more scope for novelty at the beginning of the contest, while novelty naturally decreases over time as possible space of solutions is explored and exhausted. Therefore, we settled on similarity to the current leader as a relative measure that is conditional on the current level of novelty entertained by the best entries. Code similarity was measured using the Czekanowski similarity, designed as a statistic for comparing two ecological samples in terms of proportion of overlapping species, given by

$${\mathrm{CZ}}_{\mathrm{ik}}=2\;\frac{\sum_{j=1}^{S}\text{min}(x_{\mathrm{ij}},x_{\mathrm{kj}})}{\sum_{j=1}^{S}(x_{\mathrm{ij}}+x_{\mathrm{kj}})}$$ (1)

where *CZ_ik_* is the similarity between samples *i* and *k*, *x_ij_* is the number of instances of species *j* in sample *i*, and *x_kj_* is the number of instances of species *j* in sample *k*. For our analysis, each sample corresponds to an entry, and each species is a line of code. Every entry is a set of lines of code, so the similarity between two entries is a function of the total number of lines they have in common, including reoccurring lines, relative to the sum of their lengths. Each individual contestant could thus be characterized by a distribution of leader similarities—the novelty introduced by an individual is therefore given by the distribution of dissimilarities (i.e., 1– *CZ_ik_* for each entry).

Performance of an entry was simply characterized as whether that entry became, on submission, the leader in its contest (i.e., achieved the best score at the time of its submission and thus improved the overall score). Extending this to the contestant level allowed us to quantify how many of each contestant’s entries improved upon the current leader. To test the link between social-information use at the individual level and contestant performance, we fitted a model that predicts whether a contestant ever became a leader or not (within a contest) as a function of that contestant’s social-information use. We used both the mean and the range of the distribution of similarities between a contestant’s entries and the current leader as measures of copying and exploration around the population consensus. Thus, we fitted a generalized linear mixed model with a binomial error distribution. The predicted outcome of the model was whether an individual was a leading contestant, and the dependent variables were the mean and range of the distribution of similarities between that individual’s submissions and the current leader at the time of submission.

An additional independent variable accounts for the fact that some contestants were better players overall. Thus, the model also included an average performance measure for each contestant as a fixed effect. We used the difference in score between the current leading entry and each specific entry as a continuous, relative measure of performance at the entry level, which takes into account the steady improvement in score. This score difference is positive for entries that improved the overall score, and negative for most entries—a large negative difference indicating a particularly unsuccessful entry. We rescaled this increment within each contest so it fell between −1 and 1 according to Eq. 2

$$I\prime =\text{sign}(I)\frac{I-I_{\text{min}}}{I_{\text{max}}-I_{\text{min}}}$$ (2)

where *I* is the original increment value, *I*_min_ and *I*_max_ are the minimum and maximum values taken by all increments, and *I*′ is the rescaled increment. We included the mean score increment for each contestant as a fixed effect in the model. The model also includes contest as a random effect to account for inherent differences in performance and similarity introduced by different tasks in different contests. Therefore, the model specification was

$$\begin{array}{c} {\mathrm{Leader}}_{\mathrm{ij}}\sim \text{Binomial}(1,p_{\mathrm{ij}})\\ \text{logit}(p_{\mathrm{ij}})=\alpha +\beta_{1}\times {\mathrm{MeanIncrement}}_{\mathrm{ij}}+\beta_{2}\times {\mathrm{MeanSimilarity}}_{\mathrm{ij}}+\beta_{3}\\ \times {\mathrm{RangeSimilarity}}_{\mathrm{ij}}+a_{i}\\ a_{i}\sim N(0,\sigma_{a}^{2}) \end{array}$$

where leader*_ij_* is the probability of contestant *j* in contest *i* to become a leader, and *a_i_* estimates the random effect corresponding to contest *i*. All models were implemented in R, using the lme4 package (*42*).

In the context of the MATLAB contests, being a leader was a highly prized achievement and a principal motivator for contestants. Here, “leading entries” and “leaders” have the broader significance of improving the overall score at the population level. As a result of the considerable copying taking place, most entries scored just below the current leader, making those entries that did surpass the leader even more salient. This pattern extended to the contestant level: Most contestants, including leaders, had a mean increment value just below zero. Leaders whose mean increment value exceeded zero generally submitted a small number of entries (one or two), while many leaders had a negative increment value because they submitted both leading and nonleading entries. For these reasons, whether a contestant was a leader or not is a more meaningful measure of performance than mean increment (or other continuous measures of performance), although we also fitted an additional linear model similar to the above, in which we use mean increment as the outcome variable

$$\begin{array}{c} {\text{MeanIncrement}}_{\mathrm{ij}}=\alpha +\beta_{1}\times {\text{MeanSimilarity}}_{\mathrm{ij}}+\beta_{2}>\times {\text{RangeSimilarity}}_{\mathrm{ij}}+a_{i}\\ a_{i}\sim N(0,\sigma_{a}^{2}) \end{array}$$

### Measuring individual influence

To investigate whether individuals formed reputations that affected how they were copied, we needed to establish the extent to which an individual was copied throughout the contest. While we used similarity as a proxy for copying, this does not exclude the possibility that the two entries are related through copying via a third entry they both copied. As quantifying indirect copying is impossible in this context, we devised a measure that we call influence that attempts to capture how much of an entry a population picked up following the entry’s submission.

Influence was calculated as the average similarity between an entry and subsequent entries in that contest. To control for the situation in which a contestant is working on a solution and submits a series of very similar solutions to each other, we only took into account subsequent entries submitted by other contestants. This excludes self-similarity as an explanation for high influence. The influence of the entries submitted at the beginning of the contest will naturally be lower than the influence of the entries submitted toward the end, purely because the number of subsequent entries is higher for the entries submitted at the beginning of the contest, which translates into a higher number of entries that could potentially be dissimilar to these initial entries. Therefore, we divided this average similarity by a number indicating the order of the entry into the competition, ranging from 1 for the first entry to the total number of entries in the contest for the last. We used the order of submission rather than the raw time point of submission to control for variation in the rate of submission across the duration of the competition (although the results hold when using raw time point as a normalizing factor). As mentioned above, in this analysis, we only included data starting with day 3, when participants had full access to everybody else’s entries; therefore, this timestamp never actually took the value 0. To correct for the skew introduced by the difference in magnitude between similarity and this measure of time, we used a log transformation of the influence measure—this skew correction was used for both measures of time, raw time point, and entry order. Thus, influence was given by

$$\text{Influence}=\text{log}(\frac{\text{mean similarity}}{\text{entry order}}+10^{-5})$$ (3)

Last, this influence measure was rescaled between 0 and 1 using the same form as Eq. 2 to make comparison across contests possible. Influence is therefore a continuous measure of subsequent-entry similarity for each entry that indicates how much a given solution, once introduced, is used by others in the population. This measure does distinguish between the initial innovator and the following copiers purely because innovators have precedency and therefore a higher number of entries that can potentially copy them, but it does not completely discount copiers as completely lacking influence on the population outcomes—copiers deserve credit, too, for recognizing a successful solution and popularizing it, thus influencing the population repertoire.

### Within-contest influence

To test whether leading contestants had a higher influence than nonleading contestants in either or both their leading and nonleading entries, we fitted a linear mixed model with the influence of each entry as the dependent variable. The influence was predicted as a function of a factor with three levels that specified whether (i) the entry took the lead and was submitted by a leading contestant, (ii) the entry did not take the lead but was submitted by a leading contestant, and (iii) the entry did not take the lead and was submitted by a nonleading contestant. The intercept baseline was set to group 3, the entries that did not take the lead and were submitted by nonleading contestants. The model also included the entry’s score increment as a fixed effect, because better-performing entries can be expected to have higher influence irrespective of the contestant who submitted them. This allows us to compare between prestige-bias, here measured as how much more influence entries submitted by leading contestants have, and payoff-bias, measured by the score increment of the entry. The contestant and the contest were included as random effects, with contestant nested within contest, to account for the fact that each contest might be characterized by a different average level of copying and that within each contest some contestants might have generally higher influence independent of their leader status. Therefore, the model specification was

$$\begin{array}{c} \mu_{\mathrm{ijk}}=\alpha +\beta_{i}+\gamma_{\mathrm{ij}}+\beta_{1}{\text{Increment}}_{k}+\beta_{2}{\text{ContestantFactor}}_{k}\\ \beta_{i}\sim N(0,\sigma_{1}^{2});\gamma_{\mathrm{ij}}\sim N(0,\sigma_{2}^{2})\\ {\text{Influence}}_{\mathrm{ijk}}\sim N(\mu_{\mathrm{ijk}},\sigma_{3}^{2}) \end{array}$$

for each entry *k* submitted by contestant *j* in contest *i*, where β*_i_* indicates the random effect corresponding to contest *i*, and γ*_ij_* captures random effects corresponding to contestant *j* in contest *i*.

The predictor of interest here was the contestant factor. We expected leading entries submitted by leading contestants to have a significantly higher influence than entries submitted by nonleading contestants. However, if prestige bias was operating, we also expected greater influence of nonleading entries submitted by leading contestants compared to entries submitted by nonleading contestants.

### Cross-contest influence

Some individuals participated in multiple contests, which gave us the opportunity to investigate whether individuals performed consistently across different problems or whether the variation between contest problems somehow breaks down these individual characteristics. This was tested using a similar mixed linear model as for within-contest influence. In this context, however, the predictor of interest was a factor that specified whether the entry took the lead, whether the contestant submitting the entry was ever a leader in the same contest, or whether the contestant was ever a leader in a different contest. This factor had six levels: (i) nonleading entry submitted by a nonleading contestant who was never a leading contestant in any other contest, (ii) nonleading entry submitted by a nonleading contestant who was a leading contestant in a different contest, (iii) nonleading entry submitted by a leading contestant who was not a leading contestant in a separate contest, (iv) nonleading entry submitted by a leading contestant who was also a leader in a different contest, (v) leading entry submitted by a leading contestant who was not a leader in a different contest, and (vi) leading entry submitted by a leading contestant who was also a leading contestant in a different contest. As before, we included score increment as a fixed factor, and contest and contestant identity as random factors—in this case, the model included a random effect for contest nested inside the random effect for contestant, as contestant identity explained more variation than contest identity. To capture within-participant variation adequately and to ensure methodological validity, we chose to examine individuals who participated in at least three contests, giving a sample size of 96 repeat contestants, of the total of 1416 unique contestants overall.

This allowed us to establish whether entries had more influence when submitted by a leading contestant, independent of how well they scored. Crucially, this analysis also allowed us to establish if entries had more influence when submitted by a contestant that was a leader in a different contest (i.e., if reputations carry across contests, as predicted if prestige bias is important). If entries that do not take the lead, submitted by contestants who do not become leaders in the same contest, but who had been leading contestants in a different contest still have higher influence than entries submitted by nonleading contestants both within and across contests, it would mean that the leadership reputation at the individual level was maintained across contests, evidence of prestige bias.

## Supplementary Material

aaz0286_SM.pdf

## SUPPLEMENTARY MATERIALS

Supplementary material for this article is available at http://advances.sciencemag.org/cgi/content/full/6/23/eaaz0286/DC1
