# Supplementary material for: Flexible learning, rather than inveterate innovation or copying, drives cumulative knowledge gain
Source: Sci Adv. 2020 Jun 5;6(23):eaaz0286. doi: 10.1126/sciadv.aaz0286 (PMC7274806; doi:10.1126/sciadv.aaz0286)
Supplement: aaz0286_SM.pdf [file aaz0286_SM.pdf]

Science Advances
AAAS

advances.sciencemag.org/cgi/content/full/6/23/eaaz0286/DC1

Supplementary Materials for

Flexible learning, rather than inveterate innovation or copying, drives cumulative knowledge gain

Elena Miu*, Ned Gulley, Kevin N. Laland, Luke Rendell

*Corresponding author. Email: elena.miu@gmail.com

Published 5 June 2020, *Sci. Adv.* **6**, eaaz0286 (2020)
DOI: 10.1126/sciadv.aaz0286

This PDF file includes:

Figs. S1 to S6
Tables S1 and S2
Example problem

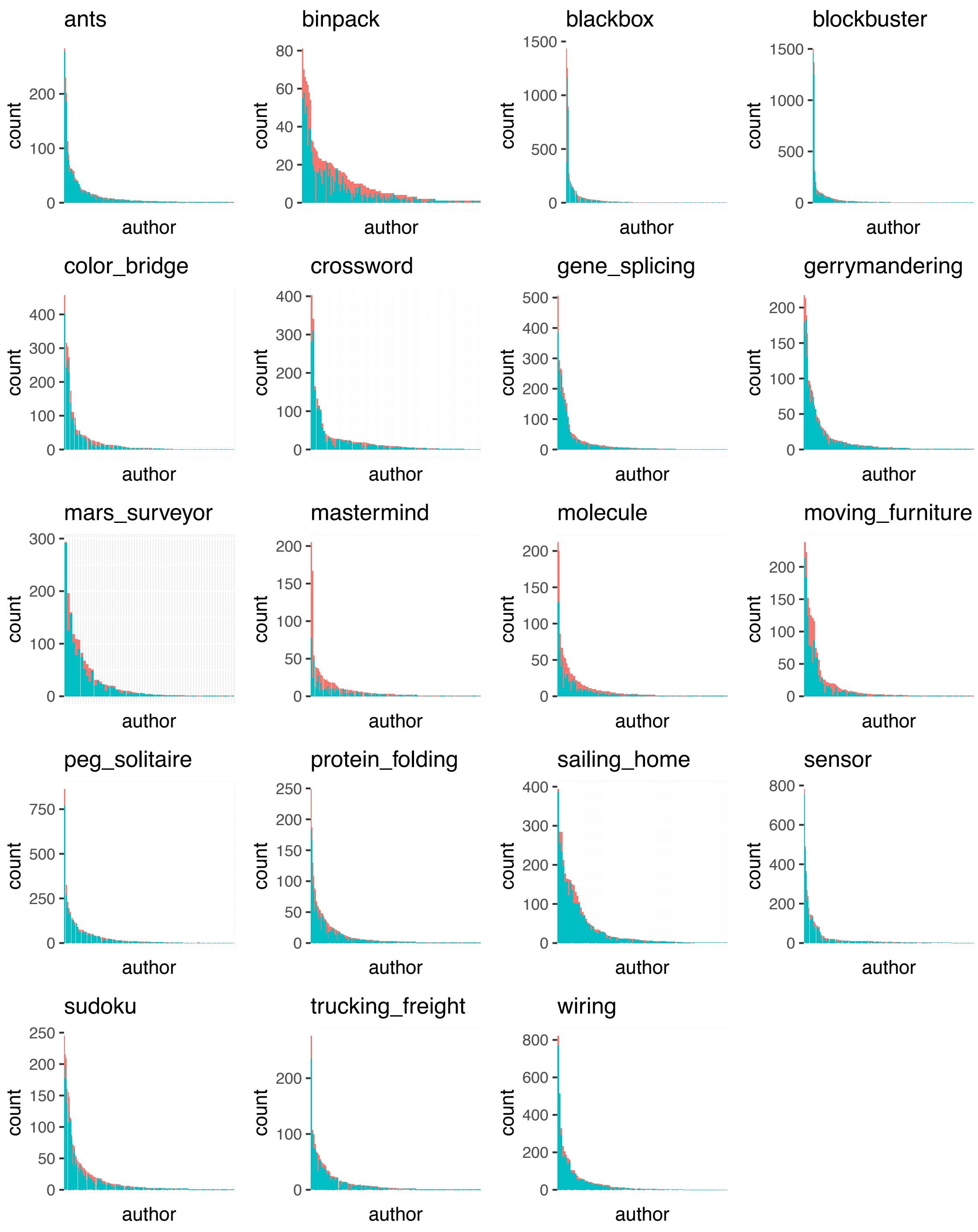

Fig. S1
Number of entries submitted per author.
Number of total entries (red indicates failed entries, blue indicates passed entries) per author for each of the 19 contests

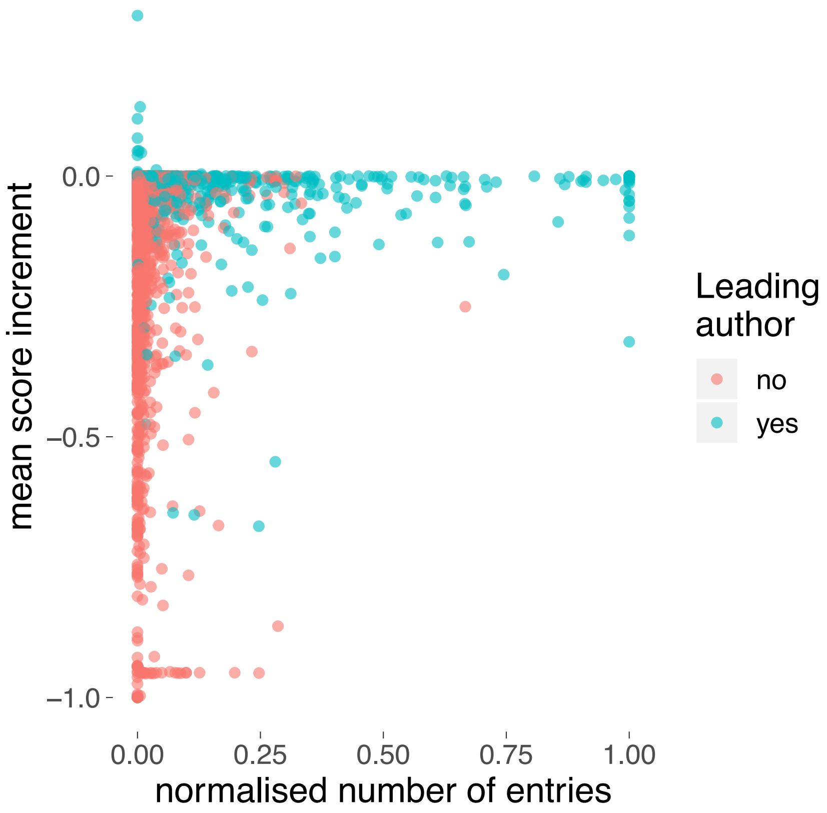

Fig. S2

Performance as a function of number of entries per author.
Average score increment relative to the current leader, for each author in each contest, as a function of the number of entries, rescaled in [0,1]. Blue points indicate leading authors (i.e. authors that took the lead in any contest), red dots indicate non-leading authors (i.e. authors that never took the lead). Authors who achieved leading status tend to be highly active (in blue, top right-hand of the graph), while barely any authors who were not active achieved leading status (in red, bottom left-hand of the graph).

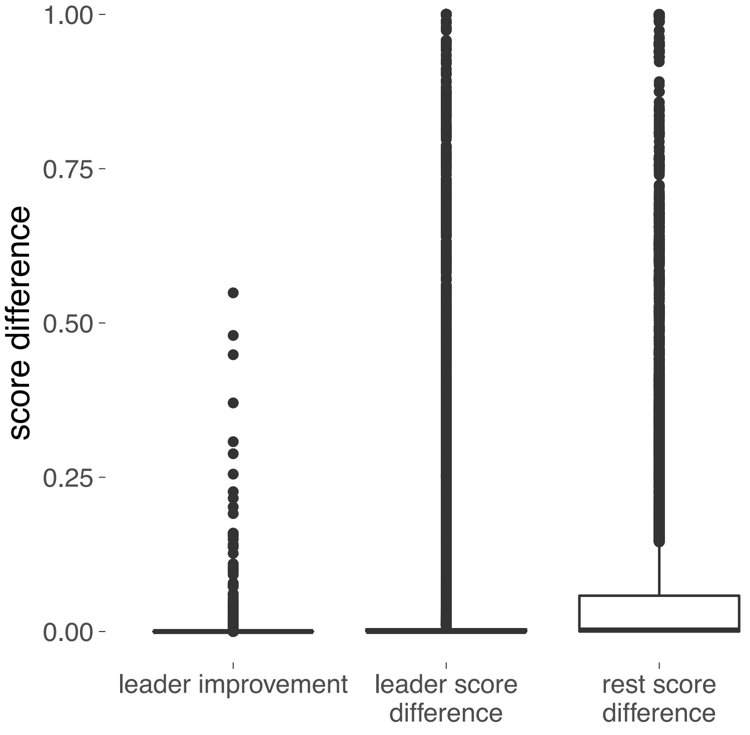

Fig. S3

Positive and negative score differences as a function of leadership.

Distribution of positive score differences submitted by leading authors (left), negative score differences submitted by leading authors (centre), and negative score differences submitted by non-leading authors (right).

Fixed effects	Estimate	Std. Error	z-value	95% confidence interval
**(Intercept)**	-0.204	0.0205	-9.987	-0.245 – -0.163
**Mean leader similarity**	0.210	0.0104	20.053	0.189 – 0.230
**Range leader similarity**	0.0165	0.009	1.675	-0.002 – 0.036

Table S1
The effect of social learning and exploration on performance.
Perfomance is measured as normalized mean increment. Results from GLMM: *MeanScoreDifference ~ MeanLeaderSimilarity + RangeLeaderSimilarity + (1|Contest).* Predictors are standardized – similarity ranges theoretically between 0 and 1, and score difference between -1 and 1.

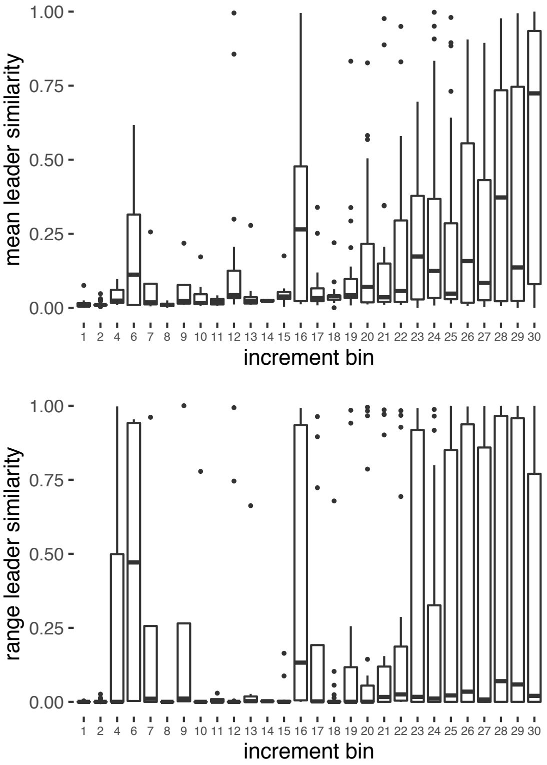

Fig. S4
Leader similarity mean and average by performance.
Distributions of average leader similarities and distribution of leader similarity ranges as a function of average contestant performance. Performance was measured as normalized score difference, and binned for visualization purposes.

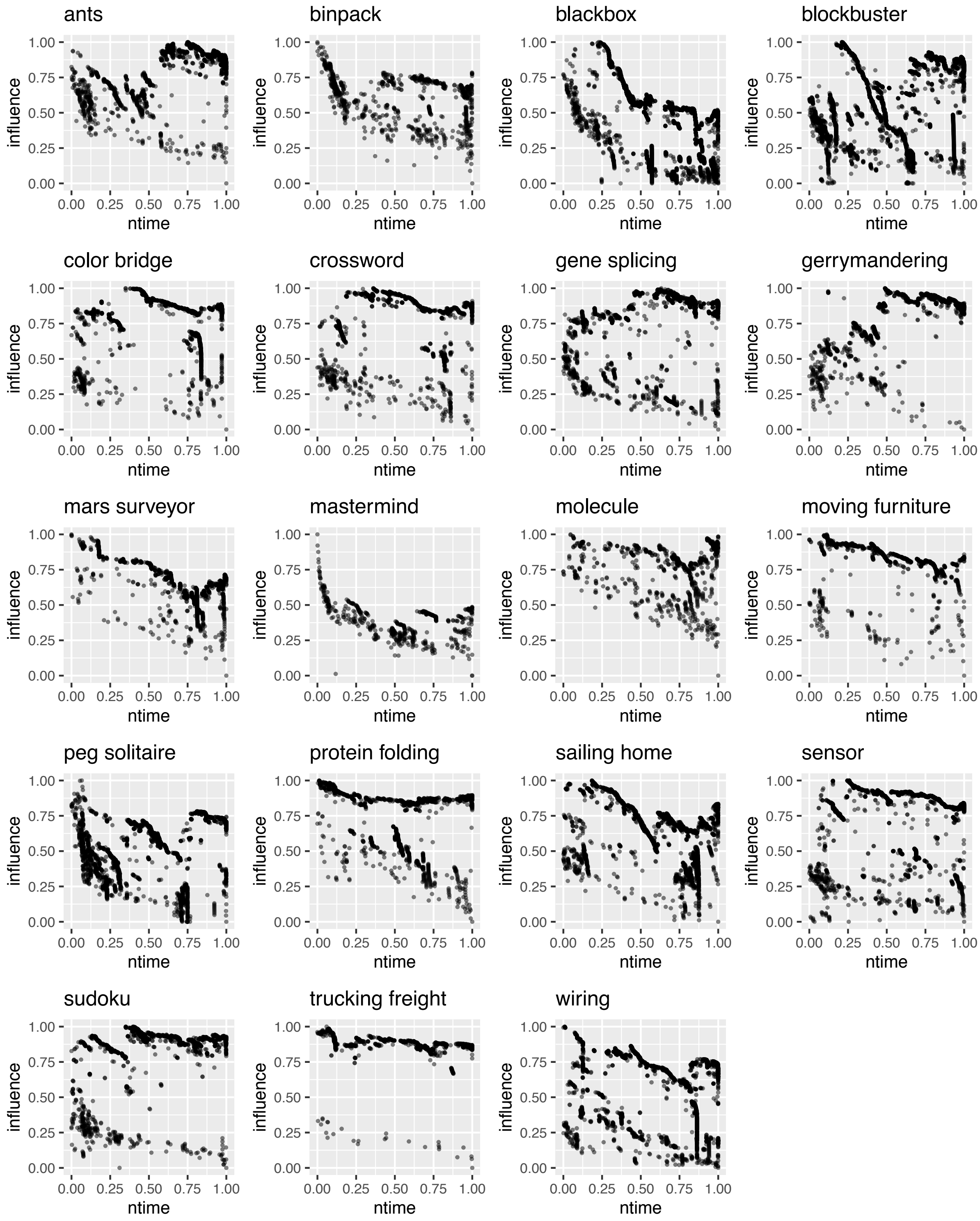

Fig. S5

Influence over time.

Influence for all passed entries submitted from day 3 onwards in all 19 contests. The y-axis represents the influence (log-transformed normalized average similarity between each entry and the subsequent entries submitted by other contestants), and the x-axis plots time, normalized between 0 and 1.

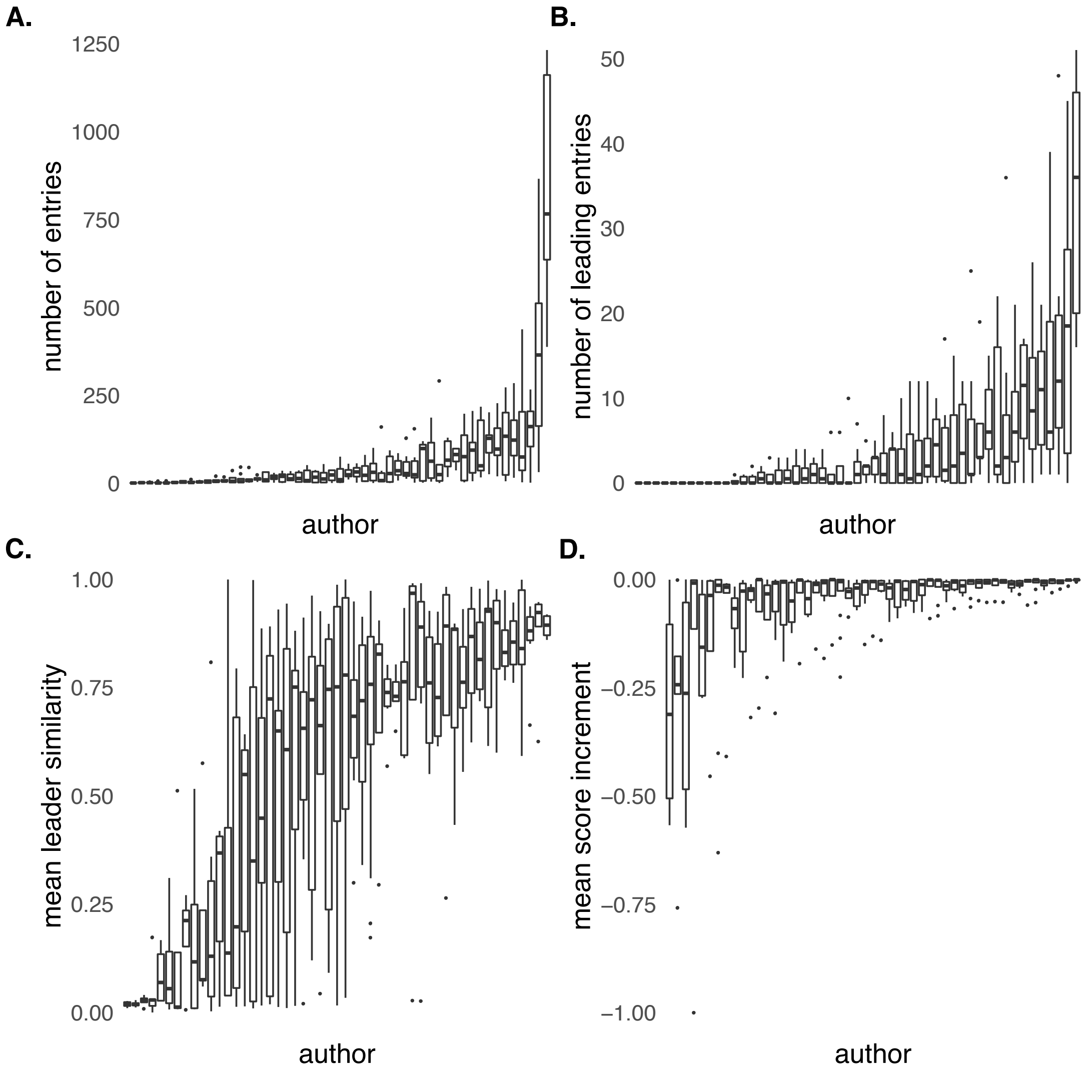

Fig. S6

Author information across contests.

Per author cross-contest distribution of the number of entries (A), the number of leading entries (B), per-contest average score increment relative to the current leader (C), and per contest average similarity to the current leader (D). Each boxplot represents the distribution of the number of entries for (A) and (B), and average values for (C) and (D) for an author across all the contests the author participated in

Name	Date	Number of players	Number of entries	Number of passed entries	Total number of lines
**Ants**	May 2005	167	2206	1972	18497
**Binpack**	Dec 1998	138	1455	877	6666
**Blackbox**	Nov 2006	170	6367	4600	85754
**Blockbuster**	Apr 2006	183	5922	5150	26546
**Color Bridge**	Nov 2009	117	2837	2270	14684
**Crossword**	Apr 2011	94	2280	1847	18362
**Gene Splicing**	Nov 2007	136	3285	2687	38760
**Gerrymandering**	Apr 2004	169	2392	2038	32749
**Mars Surveyor**	Jun 1999	63	1647	1371	10321
**Mastermind**	Sep 2001	123	1138	511	6322
**Molecule**	May 2002	154	1631	977	8286
**Moving Furniture**	Nov 2004	109	1834	1270	14282
**Peg Solitaire**	May 2007	119	3914	3426	19428
**Protein Folding**	Nov 2002	202	2437	1881	11901
**Sailing Home**	Nov 2010	98	3616	3175	17183
**Sensor**	Apr 2010	182	4814	4232	21503
**Sudoku**	Nov 2005	186	3061	2439	22778
**Tracking Freight**	Apr 2003	129	1661	1363	7369
**Wiring**	Apr 2008	106	4166	3707	92181

Table S2

Contest information.

Dates and sample size information for all 19 contests included in the dataset

Example problem: Peg Solitaire, May 2007

This contest is based on a simple peg jumping game. In a typical game of Peg Solitaire, the board contains pegs (sometimes marbles) and at least one empty space. Pegs can be removed by jumping over them with another peg, and the aim is to remove as many pegs with a combination of jumping moves.

This implements an extended version of the original Peg Solitaire game, in which the pegs carry points, and the goal is to jump pegs in order to make the score as low as possible. This may mean it is not necessary to remove all the pegs. Each peg has a value, or weight. A move consists of one peg jumping over and thereby removing another peg. A "jump" is a horizontal or vertical move in which one peg passes over exactly one other peg and comes to rest on an empty space. Diagonal jumps are not permitted. There is a reward for every peg removed from the board according to its weight, and a penalty for each jump according to the weight of the jumping peg. The score is therefore the difference between the value of the peg being jumped over and the jumping peg. Therefore a good score can be achieved by jumping with a low value peg over a high value peg; the bigger the difference between the values of the two pegs, the better the score. If, however, a high value peg is used to jump over a low value peg, the score decreases.

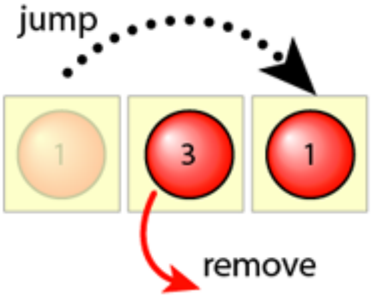

A peg with weight 1 jumps over a peg with weight 3. The reward is 3 and the penalty is 1, therefore the value of the move is 2. If several jumps in a row can be performed using the same peg, the penalty only has to be paid once.

In more detail:

- Each peg has a weight, which is always positive.
- The board is a matrix. Each positive number indicates a peg, zeros indicate empty squares, and negative numbers indicate off limits squares outside of the board.
- Every move is a four-element row vector with the format [from_row from_column to_row to_column]
- The code must return a four-column move matrix in which each row represents one move. This matrix can have any number of rows between 0 and (numpegs – 1). Any number of rows exceeding this are ignored.
- The value of each move is the sum of the removal bonus and the jumping penalty.
- Consecutive moves by a single peg only incur one jumping penalty.
- The score starts at a high value (the sum of all the peg weights). After each move, the point value of that move is subtracted from the score. The goal is to minimize the score.
- An invalid move does not generate an error – the board remains unchanged and the jumping penalty is still paid.

The overall score of an entry is a combination of three factors:

- result – the average score across all game boards
- runtime – how fast the code runs

– complexity – cyclomatic complexity - a measure of the number of independent paths through a program's source code. Typically, as this number gets higher, the program becomes less transparent and more difficult to understand.

The final score is calculated according to the equation:

$$score = k_1 * result + k_2 * e^{k_3 * runtime} + k_4 * \max(complexity - 10, 0)$$

The goal is to minimize all three factors. The lowest overall score at the end of the contest wins. An entry is disqualified if it has a runtime over 180 seconds.
